# CAR-T Cell Therapy: Mechanism, Management, and Mitigation of Inflammatory Toxicities

**DOI:** 10.3389/fimmu.2021.693016

**Published:** 2021-06-18

**Authors:** Joseph W. Fischer, Nirjal Bhattarai

**Affiliations:** Division of Cellular and Gene Therapies, Office of Tissues and Advanced Therapies, Center for Biologics Evaluation and Research, U.S. Food and Drug Administration, Silver Spring, MD, United States

**Keywords:** CAR-T cells, inflammation, toxicities, CRS, neurotoxicity

## Abstract

Engineered T cell therapies such as chimeric antigen receptor (CAR) expressing T cells (CAR-T cells) have great potential to treat many human diseases; however, inflammatory toxicities associated with these therapies present safety risks and can greatly limit its widespread use. This article briefly reviews our current understanding of mechanisms for inflammatory toxicities during CAR T-cell therapy, current strategies for management and mitigation of these risks and highlights key areas of knowledge gap for future research.

## Introduction

Although CAR-T cell therapy has been in development for more than two decades (1), recent U.S. FDA approval of one BCMA-targeting and several CD19-targeting CAR-T cell therapies for certain relapsed/refractory hematologic malignancies have energized the field (2–8). Many other CAR-T cells targeting various antigens other than CD19 are either in pre-clinical development or currently in clinical trials to treat various human diseases such as cancer, infectious diseases, autoimmune diseases, cardiac diseases etc. (9, 10). It is anticipated that the field of CAR-T cell therapy will continuously grow, and new CAR-T cells will be developed to treat previously incurable human diseases.

Based on the data obtained from clinical studies with CD19 CAR-T cell therapies, CAR-T cells can be curative in those patients who respond to the therapy; however, not all patients respond in a similar manner and challenges such as resistance and relapses have been observed (3, 11–14). Following infusion, CAR-T cells may fail to proliferate or persist leading to loss of therapeutic response (15). One of the mechanisms that can lead to loss of function is CAR-T cell exhaustion (3, 12). Strategies such as use of checkpoint inhibitors (e.g. anti-PD-1 or anti-PD-L1) to block PD-1 and PD-L1 interaction between CAR-T cells and cancer cells are being used in clinical trials to restore CAR-T cell function (15, 16). Other strategies to mitigate CAR-T cell exhaustion are also in development (17–19).

Loss or decrease in target antigen expression can also affect efficacy of CAR-T cell therapy. Antigen-null or antigen-dim cancer cells can arise *via* various mechanisms, which can contribute to resistance to CAR-T therapy (20–23). Experience with CD19 CAR-T cells suggest a significant number of patients can relapse due to loss of CD19 expression (11). Other rare events such as transduction of a tumor cell during manufacturing and subsequent CAR expression in the malignant cell resulting in resistance to CAR-T cell therapy has been also observed (24). Thus, numerous mechanisms may contribute to resistance and relapse during CAR-T cell therapy. Therefore, future studies are warranted to better understand mechanisms for resistance and relapses during CAR-T cell therapy and develop novel strategies to overcome these challenges.

CARs are synthetic receptors that are developed to interact with target cells with high specificity. Although CAR-T cells with high specificity are desirable, expression of target antigen in normal cells can lead to unwanted toxicities. For example, CD19-targeting CAR T cells effectively kill both CD19+ tumor cells and CD19+ normal B cells resulting in prolonged B cell aplasia (25). Long-term B cell aplasia can be detrimental and can lead to increased risk of infections (25). Expression of target antigen in normal cells can also pose a significant safety risk. In one study, a patient receiving an ERBB2-targeting CAR-T cell therapy died after 5 days, because the CAR-T cells unintentionally targeted lung epithelial cells that expressed low levels of ERBB2 (26). Thus, it is critical to develop strategies to specifically target diseased cells while sparing healthy cells during CAR-T cell therapy (27).

Another hallmark challenge associated with almost all CAR-T cell therapies is development of systemic inflammatory toxicities such as cytokine release syndrome (CRS) and neurotoxicity (28). CRS symptoms can include flu-like symptoms, hypotension, capillary leak, hypoxia, and severe CRS can lead to multi-organ failure. Neurotoxicity symptoms can include headaches, delirium, seizures, and cerebral edema. These symptoms usually occur within the first two weeks, but more severe cases can present within 72 hours (28). In CD19 CAR-T cell therapies, CRS severity was found to vary significantly among patients with up to 46% of severe cases observed in some studies, which typically requires admittance to the intensive care unit (2–6, 29). These inflammatory toxicities have been observed in almost all CAR-T cell clinical trials, including the FDA-approved CAR-T cell therapies Kymriah, Yescarta, Tecartus, Breyanzi and Abecma (2–7). While new CAR-T cell clinical trials are still facing these challenges, methods to diagnose and better treat inflammatory toxicities during CAR-T cell therapy are also in development (6, 30, 31).

One of the major challenges for developing effective treatment strategies for inflammatory toxicities during CAR-T cell therapy is poor understanding of mechanisms for heterogenous inflammatory response and factors that contribute to these toxicities. CAR-T cells are also being developed to treat various solid tumors; however, there are several challenges that need to be addressed to improve safety and efficacy of CAR-T cells against solid tumors (32). Furthermore, severity of inflammatory toxicities that may arise following CAR-T cell treatment against solid tumors is poorly understood. Thus, future work to understand mechanisms for inflammatory toxicities and identification of factors that contribute to these toxicities during CAR-T cell therapy against solid and liquid tumors may help in rational design of CAR-T cells that are safer and may also help in developing novel strategies to effectively manage and treat these toxicities.

## Current Understanding of Mechanisms Contributing to Inflammatory Toxicities

Inflammatory toxicities such as CRS and neurotoxicity are associated with the presence of high levels of inflammatory proteins and cytokines such as GM-CSF, IL-6, IL-1β, C-reactive protein (CRP), etc. in the serum of patients treated with CAR-T cells (33, 34). One of the major sources of these pro-inflammatory cytokines are myeloid cells that are activated during CAR-T cell therapy (35–38). Post-infusion and following target cell recognition, CAR-T cells are activated and secrete various inflammatory factors, such as GM-CSF, which can activate myeloid cells and promote the rapid production and secretion of pro-inflammatory cytokines such as IL-6 and IL-1β that contribute to inflammatory toxicities (37, 39). Since, cytokines like IL-6 and IL-1β are not primarily produced by CAR-T cells, targeting these cytokines by immunomodulatory agents may not completely prevent CRS and neurotoxicity; however, understanding mechanisms for myeloid cell activation during CAR-T cell therapy may help develop strategies that can prevent or reduce activation of myeloid cells. One such strategy to reduce myeloid activation is through reducing extraneous CAR-T cell activation that can occur from on-target/off-tumor activity (40). Thus, by targeting antigens that are expressed only in diseased cells or by implementing better strategies to reduce on-target/off-tumor activity, unwanted CAR-T cell activation may be reduced, which may help reduce bystander myeloid cell activation.

Toxicity from CAR-T cell therapies can greatly vary, which can be due to multiple factors including patient heterogeneity, baseline tumor burden, CAR-T cell dose, differences in starting material for autologous products or the CAR-T cell manufacturing process (34).Tumor burden can be predictive of toxicities as clinical studies have found that patients with a higher baseline tumor burden or treated with higher number of CAR-T cells have greater incidence of inflammatory toxicities and demonstrate poor survival compared to patients with lower tumor burden (34, 41). Other factors, such as patient microbiome, metabolome, or cytokine profile etc. may also modulate CAR-T efficacy and safety and warrant further investigation (42, 43).

Manufacturing process can also greatly affect CAR-T cell safety and efficacy (44). For example, selection of CD4 and CD8 positive T cells following apheresis during manufacturing improved CAR-T cell efficacy, but this process also resulted into CAR-T cells with increased inflammatory toxicities (45). T cell subsets or activation and/or differentiation state of T cells in the CAR-T cell product can also impact CAR-T cell safety and efficacy. CAR-T cell products containing higher numbers of stem-like memory cells or central memory have demonstrated improved expansion, persistence and efficacy (46, 47). Single-cell analysis of various CAR-T cell subsets has revealed different populations of cells contribute to different inflammatory cytokines and effector function (48). Further studies on impact of various T cell subsets present in the CAR-T cell product on safety and efficacy may help improve safety by allowing for either enrichment or depletion of specific T cell subset.

CAR-T cell design and choice of co-stimulatory signaling molecules can also impact safety and efficacy (19). First-generation CARs used a single signaling domain, which was found to have poor persistence and low efficacy (49–52). The first-generation CARs were primarily derived from murine antibodies, which may have also contributed to poor persistence and low efficacy. With the addition of a co-stimulatory domain in the second-generation CARs such as the FDA-approved CAR-T cell products, CAR-T cell efficacy was greatly improved. However, patients treated with these more active CAR-T products also experienced inflammatory toxicities (2–6). Additionally, studies have evaluated correlation between specific domains and susceptibility to inflammatory toxicities. For the five FDA-approved CAR-T cell products, they either contain the CD28 (Yescarta and Tecartus) or 4-1BB (Kymriah, Breyanzi and Abecma) as co-stimulatory signaling domain, correlation between signaling domains and toxicity has remained inconclusive in part due to differences in clinical grading scales and cancer types (19). Due to improvements in inflammatory toxicity management over recent years, it has been challenging to directly compare various studies as more recent studies treat patients earlier or even prophylactically in some cases (6). Thus, it will be important for future clinical studies to compare the safety and efficacy of new emerging CAR-T therapies with other CAR-T cells to better understand effect of various co-stimulatory domains on inflammatory toxicities.

While second generation CAR constructs are used in FDA-approved products and many more are in clinical development, some clinical studies are also assessing third generation CAR constructs that often contain both the CD28 and 4-1BB stimulatory domains (31). Although due to differences between patient population, trial design etc. results from these third-generation CAR-T cells cannot be compared with second-generation CAR-T cells, results from early trials have been encouraging and have shown that third-generation CAR-T cells can achieve higher levels of activation without increasing the frequency and severity of inflammatory toxicities (31, 53). The increased activation without increased toxicity may be due to the earlier use of immunomodulators, rapid CAR-T exhaustion, differences in baseline tumor burden or other unknown mechanisms that warrant further investigation Data from a multi-arm clinical trial using either second-generation or third-generation CAR-T cells may also provide additional insights into this observation. As new CARs are designed and used to treat various diseases, it will be important to better understand how these changes will impact not only their ability to eliminate target cells, but also produce sustained effect and reduce the risk of severe side-effects.

## Current Strategies for Management of Inflammatory Toxicities and Limitations

Currently, following onset of inflammatory toxicities, treatment strategies focus on reducing overall inflammation by using corticosteroids, or inhibiting inflammatory cytokines, such as IL-6 or IL-1β, signaling pathways (28, 54). Although, administration of the anti-inflammatory therapies are typically given following onset of CRS; some studies have also used it prophylactically (30). While these treatment strategies are effective in reducing severity of these inflammatory toxicities, they do not prevent their occurrence, nor do patients uniformly respond to these interventions (28). Furthermore, immunosuppressive agents such as corticosteroids have a systemic effect, blunting immune cell responses that can impact therapeutic efficacy. Some studies suggest that the effect of corticosteroids on CAR-T cell function is likely to be minimal (30, 55); however, other studies have found it to be inhibitory (56). These differences may be due to the timing, duration and dose of steroids used in these studies. In addition, corticosteroid resistant inflammatory toxicities have been observed during CAR-T cell therapy resulting in multi-organ failure and death (57).

Pro-inflammatory cytokines such as IL-6 and IL-1β are commonly associated with inflammatory toxicities during CAR-T cell therapy and these cytokines are significantly elevated in the blood following CAR-T cell infusion (35). These myeloid-derived cytokines activate other immune cells including themselves (35). Thus, anti-IL-6 or anti-IL-1β therapy could prevent the positive-feedback loop of myeloid activation and help reduce CRS severity. Currently tocilizumab, an IL-6 receptor antagonist, is the only FDA-approved therapy for treating CAR-T cell-associated CRS (58). Additionally, it has been also used as a preventative treatment, including patients with high tumor burdens, as it does not seem to affect CAR-T cell function and can mitigate the initial severity of CRS (30, 59). While tocilizumab has shown promise in resolving CRS severity, it has many limitations. For example, tocilizumab has been found to be less effective in resolving neurotoxicity symptoms (60, 61). This is likely because tocilizumab is a large antibody molecule, which is unable to cross the blood brain barrier (BBB) (62). Treatment with tocilizumab can also be clinically ineffective in up to 30% of patients (58), and tocilizumab-refractory CRS can develop in some patients (57, 63). Since tocilizumab targets the IL-6 receptor and not the cytokine, its effect in some patients may be reduced due to the presence of soluble IL-6 receptor. Further studies need to evaluate the impact of other therapies on CAR-T cell associated inflammatory toxicities, such as siltuximab that binds to the IL-6 cytokine and removes it from circulation (64). Furthermore, preclinical studies have shown that IL-6 deficient mice still develop CRS symptoms when treated with CAR-T cells (65) suggesting targeting IL-6 alone may not be sufficient in all patients and additional therapeutic strategies are needed to treat both CRS and neurotoxicity.

One such inhibitor that may help treat CRS and neurotoxicity when used in in conjunction with IL-6 inhibitors is anakinra. Anakinra is an antibody-based IL-1 receptor (IL-1R) antagonist, which is an FDA-approved drug for treating patients with rheumatoid arthritis and other inflammatory diseases (66). While the use of anakinra for treating CAR-T cell induced toxicities requires additional clinical trials, this therapy may be effective in treating both CRS and neurotoxicity (54, 67). Previous work has found that anakinra and IL-1β can traverse the BBB (33, 36, 68), and in preclinical studies anakinra reduced both CRS and neurotoxicity (35, 36). However, similar to tocilizumab, blocking IL-1β signaling by anakinra may not completely treat inflammatory toxicities as IL-1R deficient mice also developed CRS symptoms following CAR-T cell infusion (65). Together, these data suggest that inhibition of inflammatory cytokines such as IL-6 and IL-1β may not be sufficient to treat inflammatory toxicities in all patients. Furthermore, the effect of these agents on bystander immune responses that also contributes to CAR-T cell efficacy is unknown. Future studies are required to better understand mechanisms contributing to these inflammatory toxicities during CAR-T cell therapy, which may aid in better management of these toxicities. It is also important to note that while current management strategies have several limitations, CAR-T cell therapy has saved many lives. Thus, current toxicity management strategies should continue to be used with CAR-T cell therapy until better management or preventive strategies are identified.

## Current Strategies to Prevent Inflammatory Toxicities During CAR-T Cell Therapy

In response to the toxicities that accompany CAR-T cell therapy, research has focused on rational CAR design and genetic manipulation of T cells in order to prevent inflammatory toxicities. New CAR constructs have been designed to contain additional domains to improve CAR-T cell activity (69). These multi-CARs increase the specificity through either requiring recognition of two antigens on the cells surface (bispecific CAR) or requiring the absence of an antigen (inhibitory CAR) (70–72). The increase in specificity can reduce unwanted CAR-T cell activation by reducing on-target/off-tumor activity, which may result into lower levels of inflammatory factors released by CAR-T cells.

Other methods are also in development to increase on-target activity. Expression of chemokine receptors like CCR4, CXCR1, or CXCR2, improved anti-tumor activity by increasing CAR-T cell localization to tumors (73, 74). Increased cellular localization to tumors may also help reduce on-target/off-tumor activation of CAR-T cells and peripheral myeloid cells. Additionally, CARs can include molecular switches that can be controlled post-infusion to modulate the level of CAR-T activation (69). While these strategies can help mitigate side-effects by reducing overall activity, they do not directly inhibit the development of inflammatory toxicities.

Use of small molecule inhibitors to prevent inflammatory cytokines signaling may also help reduce severity of inflammatory toxicities in patients. For example, Bruton’s tyrosine kinase inhibitor (BTKI) has been shown to reduce production of the inflammatory cytokine IFN-gamma from both CAR-T cells and tumor cells (75). BTKIs are FDA-approved drugs and are frequently used to treat hematological cancers prior to CAR-T cell therapy (6, 76, 77). One study found that pre-treatment with Ibrutinib (BTKI), was able to effectively reduce CRS severity (78), and this strategy is currently being evaluated in an ongoing clinical trial (NCT03960840). It has been also proposed that BTKIs reduce CRS severity through reducing the expression of inflammatory cytokines, such as IL-6 and GM-CSF (75). Although these early findings are promising, the effect of BTKIs on safety and efficacy of CAR-T cell therapies needs to be further studied.

Another small molecule, metyrosine, have been shown to prevent inflammatory toxicities through inhibiting catecholamine production (38). Catecholamines (e.g. dopamine) are secreted by CAR-T cells and act as an endogenous immunomodulator by inducing expression of various cytokines, including IL-6 and IL-8 (79). In an *in vivo* preclinical study, prophylactic treatment of metyrosine significantly reduced inflammatory cytokine levels without affecting efficacy (38). Metyrosine is an antihypertensive drug used to treat pheochromocytoma (80). Like metyrosine, atrial natriuretic peptide also inhibited catecholamines and reduced CRS-associated cytokine production from CAR-T cells (38). Since catecholamines have previously been linked to neurotoxicities (81), inhibition of catecholamines may help reduce both CRS and neurotoxicity.

Modulation of post-transcriptional processes have been also studied as a potential strategy to reduce inflammatory cytokines during CAR-T cell therapy. JTE-607 (TO-207) is a CPSF3 inhibitor that blocks pre-mRNA processing into mature mRNA and has been shown to reduce the processing and secretion of cytokines from monocytes *in vitro* (82). This mRNA-processing inhibitor had minimal effects on the release of soluble factors from CAR-T cells, suggesting that it selectively inhibits cytokine production in monocytes (82). An early clinical study found that a single dose of JTE-607 appeared to be well-tolerated and reduced the severity of endotoxin-induced inflammation in healthy individuals (83). If this inhibitor can effectively prevent the release of multiple cytokines without effecting the mRNA processing in CAR-T cells and other healthy tissues, it could be more effective than therapies that only target a single cytokine. However, the mechanism of selectively inhibiting only cytokine mRNA processing, while not effecting other cells or transcripts, requires further research. Thus, small molecule drugs can be effective in managing or treating inflammatory toxicities during CAR-T cell therapy. However, these drugs can also affect tumor cells and impact CAR-T cell efficacy, thus, effect of small molecule drugs on tumor cells should be also carefully assessed before using these drugs for treating CAR-T cell toxicities.

Another method that has been in development to modulate inflammatory cytokine expression is through genetic manipulation of CAR-T cells. As IL-6 and IL-1β are crucial cytokines involved in inflammatory toxicities, CAR-T cells have been engineered to express and secret interleukin-1 receptor (IL-1RA), which can sequester free IL-1β in circulation (36). This approach is similar to the strategy of using anakinra, but in this strategy, IL-1RA would be expressed prior to IL-1β induction and be continuously released into the blood from CAR-T cells. A similar approach could be used with an IL-6 receptor alone or in combination with IL-1RA. Another study found that knockdown of IL-6 in CAR-T cells reduced IL-6 production from monocytes, which may be due to reduction in IL-6 positive-feedback loop (39). Thus, these various strategies to design CAR-T cells to prevent IL-6 and IL-1β signaling may help reduce severity of inflammatory toxicities during CAR-T cell therapy.

Over-expression of CD40 ligand (CD40L) on CAR-T cells increased anti-tumor efficacy through CD40L interactions with CD40 expressed on tumors (84). The over-expression of CD40L also increased expression of IL-1RA resulting into lower IL-1β expression (84). Thus, this strategy of CD40L expression in CAR-T cells may help reduce inflammatory toxicities; however, CD40L also interacts with CD40 on antigen presenting cells, which may lead to increased activation of myeloid cells (35). Thus, future studies should carefully evaluate the effect of overexpression of CD40L on CAR- T cell safety and efficacy.

While targeting inflammatory cytokines such as IL-6 or IL-1β may help reduce severity of inflammatory toxicities during CAR-T cell therapy, it does not prevent the onset of these toxicities. Since these inflammatory cytokines are not primarily produced by CAR-T cells (85), targeting CAR-T cell factors that contribute to myeloid cell activation may be more effective in preventing onset of these toxicities. GM-CSF is one of those promising factors, which is secreted by CAR T-cells following activation and activates myeloid cells but does not appear to contribute to CAR-T cell function (37, 65, 86). *In vitro* inactivation of GM-CSF in CAR-T cells reduced myeloid cell-derived inflammatory cytokines (37), and in some studies GM-CSF inhibition reduced expression of some CRS associated cytokines, such as IL-6, but not IL-1β (37, 65, 86–88). However, in animal studies, GM-CSF neutralization did not completely prevent inflammatory toxicities (86). As the mechanisms for inflammatory toxicities are likely to be very complex, it is anticipated that additional factors released by CAR-T cells contribute to these inflammatory toxicities. Thus, future studies are warranted to identify these novel inflammatory factors released by CAR-T cells to help rationally design CAR-T cells that are less toxic.

Current strategies to rationally improve CAR-T cells have primarily focused on improving its efficacy (10). One of such modifications is deletion of the NR4A transcription factors to reduce CAR-T cell exhaustion and improve efficacy (18). Although, NR4A deletion improved CAR-T cell potency in preclinical animal studies, GM-CSF was significantly upregulated over 20-fold in the NR4A knockout CAR-T cells. This suggests that while deletion of NR4A may improve CAR-T cell function, it may also significantly increase myeloid cell activation and inflammatory toxicities. Thus, multiple genetic manipulations may be required to improve overall safety and efficacy of CAR-T cells. Genome editing tools have greatly improved in recent years and can effectively knock-out, modify, and insert genes within primary cells. With these improvements, it has become possible to incorporate a variety of modifications to T cells during CAR-T cell manufacturing. As new genetic modifications are introduced to improve CAR-T cell safety and efficacy, the effect of these modifications should be carefully evaluated, and a comprehensive risk assessment should be performed prior to initiation of clinical studies.

Other immune cells such as natural killer (NK) cells that express CAR (CAR-NK) are also in development (89). In a phase I clinical study, anti-CD19 CAR-NK cells demonstrated a potent anti-tumor response without causing any severe inflammatory toxicities (90). Although, additional studies will be required to further corroborate safety and efficacy of CAR-NK cells, these early data suggest that CAR-NK cells may be safer than CAR-T cells. Furthermore, future studies of CAR-NK cells and characterization of mechanism contributing to lower inflammatory toxicities during CAR-NK cell therapy may also provide insights into mechanism for inflammatory toxicities during CAR-T cell therapy and aid in rationally designing CAR-T cells that are safer and effective.

## Conclusions and Future Perspectives

**Figure 1:**
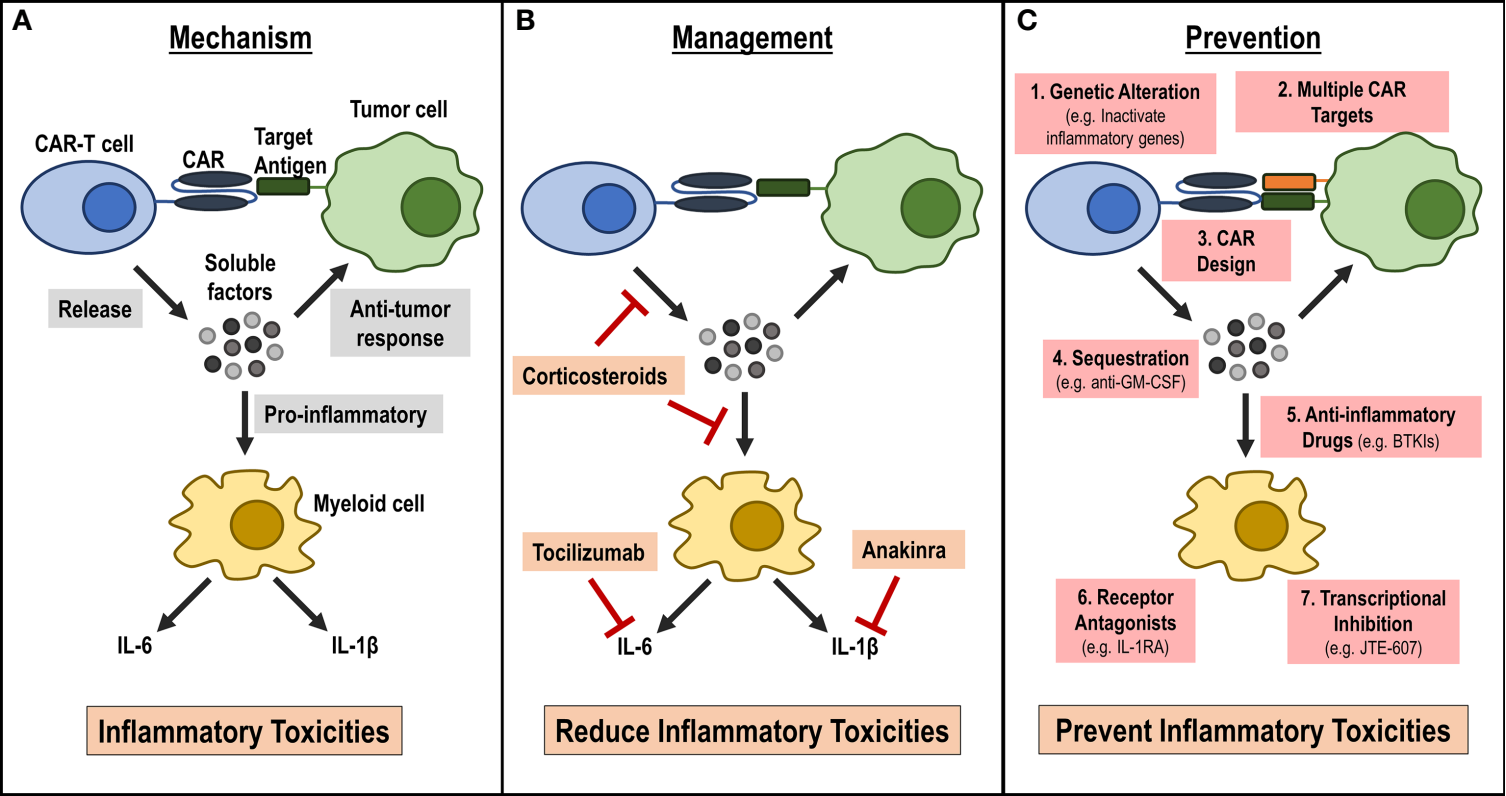
Current understanding of mechanism for inflammatory toxicities during CAR-T cell therapy, current management strategies and future strategies for prevention. **(A)** Mechanism: Activated CAR-T cells release soluble factors upon CAR engagement with target antigen. These soluble factors can aid in the anti-tumor response, or they can activate bystander myeloid cells. Activated myeloid cells secrete inflammatory cytokines, such as IL-6 and IL-1β, that lead to the inflammatory toxicities observed in patients infused with CAR-T cells. **(B)** Management: Current management strategies focus on reducing inflammatory cytokines or cytokine signaling pathways by either using anti-inflammatory drugs, such as corticosteroids that may reduce inflammatory cytokines release by CAR-T cells or myeloid cells or by targeting specific cytokine receptors, such as IL-6R by tocilizumab and IL-1R by anakinra. **(C)** Prevention: New approaches that focus on preventing the onset of CRS. These strategies include: 1) modifying CAR-T cells during manufacturing such as genetic alterations to inactivate inflammatory genes, 2) designing CARs with novel domains that are less inflammatory, 3) targeting multiple antigens on tumors to reduce on-target off-tumor activation, 4) inhibiting pro-inflammatory CAR-T cell factors using antibodies (e.g. anti-GM-CSF), or inhibiting myeloid cell activation by 5) using small molecule inhibitors (e.g. BTKI), 6) expressing inflammatory cytokine receptor (e.g. IL-1R) on CAR-T cells or 7) using myeloid cell-specific transcriptional Inhibitor (e.g. JTE-607).

As CAR-T cell therapies are being developed to treat a wide variety of human diseases, it is critical to understand inflammatory toxicities associated with these therapies and develop strategies to effectively manage or prevent these adverse events (**Figure 1**). While current treatment strategies help reduce the severity and duration of inflammatory toxicities, there are several limitations of these current strategies. Additionally, current management strategies can have heterogeneous response in patients, can be toxic and are expensive. Furthermore, they do not target the underlying cause of inflammatory toxicities during CAR-T cell therapy. Understanding mechanisms contributing to inflammatory toxicities during CAR-T cell therapies will help to greatly improve safety of these therapies by helping develop less toxic CAR-T cells during manufacturing and by developing better treatment strategies for patients who develop these toxicities (**Figure 1**). As the CAR-T cell field grows, it is anticipated that the CAR-T cell design and manufacturing process will be more complex. Thus, it is critical that future studies address these underlying challenges in an urgent manner so that the benefit of this effective therapy in treating numerous human diseases can reach to widespread population.

## Author Contributions

All authors contributed to the article and approved the submitted version.

## Conflict of Interest

The authors declare that the research was conducted in the absence of any commercial or financial relationships that could be construed as a potential conflict of interest.
